# Endoscopic submucosal dissection with an additional working channel (ESD+): a novel technique to improve procedure time and safety of ESD

**DOI:** 10.1007/s00464-020-07808-w

**Published:** 2020-07-16

**Authors:** Richard F. Knoop, Edris Wedi, Golo Petzold, Sebastian C. B. Bremer, Ahmad Amanzada, Volker Ellenrieder, Albrecht Neesse, Steffen Kunsch

**Affiliations:** Department of Gastroenterology and Gastrointestinal Oncology, University Medical Center Goettingen, Georg-August-University, 37075 Göttingen, Germany

**Keywords:** Endoscopic submucosal dissection (ESD), ESD+ technique (ESD+), Additional working channel (AWC), Endoscopic mucosal resection (EMR), EMR+, RESECT+, Animal model, EASIE-R model

## Abstract

**Background and aims:**

A new external additional working channel (AWC) was recently introduced by which endoscopic submucosal dissection (ESD) can be converted to a technique termed “ESD+ ”. We aim to systematically evaluate this novel technique in flat gastric lesions and compare it to classical ESD.

**Methods:**

The study was prospectively conducted in a pre-clinical ex vivo animal model (EASIE-R simulator) with porcine stomachs. Prior to intervention, we set standardized lesions measuring 3 cm or 4 cm in antegrade as well as in retrograde positions.

**Results:**

Overall, 64 procedures were performed by an experienced endoscopist. Both techniques were reliable and showed en bloc resection rates of 100%. Overall, ESD+ reduced time of procedure compared to ESD (24.5 vs. 32.5 min, *p* = 0.025*). Particularly, ESD+ was significantly faster in retrograde lesions with a median of 22.5 vs. 34.0 min in 3 cm retrograde lesions (*p* = 0.002*) and 34.5 vs. 41.0 min (*p* = 0.011*) in 4 cm retrograde lesions. There were 0 perforations with both techniques. In ESD+ , 1 muscularis damage occurred (3.13%) compared to 6 muscularis damages with ESD (18.75%, *p* = 0.045*).

**Conclusions:**

By its grasp-and-mobilize technique, ESD+ allows potentially faster and safer resections of flat gastric lesions compared to conventional ESD in an ex vivo porcine model. The potential advantages of ESD+ in terms of procedure time may be particularly relevant for difficult lesions in retrograde positions.

**Electronic supplementary material:**

The online version of this article (10.1007/s00464-020-07808-w) contains supplementary material, which is available to authorized users.

Many precancerous gastrointestinal lesions and early gastric cancers can successfully be treated with endoscopic mucosal resection (EMR), a well-established, safe, and cost-effective interventional endoscopic technique [1, 2]. EMR features a relatively low technical complexity, rapid procedure times and a low risk of adverse events [3]. However, EMR shows a decreasing rate of en bloc resections in larger lesions [1, 2]. Specifically, en bloc resection of large sessile or laterally spreading polyps measuring ≥ 2 cm is hardly possible via conventional EMR where en bloc resection, e.g., for colorectal lesions of this size, can only be achieved in about 30% of cases [3, 4]. Although EMR can be performed in piecemeal technique, tissue fragments are often difficult to analyze for the pathologist, and patients require frequent follow-up endoscopies due to a higher rate of incomplete resections and ultimate recurrences [5].

Classical EMR can be improved by a new external additional working channel (AWC, Ovesco Endoscopy, Tuebingen, Germany). Recently, the possibility of using the AWC was reported and the technique was termed “EMR+ ” [6–8]. First reports were also published by our group and others on its application and feasibility in humans [6, 9]. We also provided experimental data systematically evaluating the AWC in EMR [7, 8]*.* The AWC seems to extend the spectrum of EMR beyond the critical size of 2 cm, especially showing promising results concerning the rate of en bloc resections in 3 cm lesions [8]. However, in 4 cm lesions, also EMR+ reaches its inherent limits with decreasing en bloc resection rates and a relevant risk of perforations [8].

Therefore, endoscopic submucosal dissection (ESD) should be considered for lesions larger than 3 cm. Initially developed in Japan for the en bloc resection of early gastric cancers, ESD has also become an interventional endoscopic procedure in expert centers of the Western world [10–14]. ESD has also been increasingly employed in colorectal lesions and the esophagus. Particularly, ESD offers a sophisticated and anatomically reliable method for the resection of laterally spreading polyps and flat lesions ≥ 2 cm. Theoretically, ESD enables the endoscopist to achieve en bloc resections regardless of tumor size [13]. However, ESD comes along with a relevant rate of adverse events, particularly a higher rate of perforations [3]. ESD is technically complex with a considerable learning curve even for experienced endoscopists. Furthermore, it demands more resources, time, and costs.

It is worthwhile that ESD is optimized by additional endoscopic devices which need to be developed and clinically implemented. There have already been several approaches of enhancing ESD by counter-traction devices, e.g., with the EndoLifter (Olympus, Tokyo, Japan) [15]. The EndoLifter consists of a transparent hood and a bracket to which a grasping forceps is attached [15]. Following and advancing this principle, conventional ESD can be augmented by the AWC mounted on a standard endoscope facilitating a combination of grasper and ESD knife, ESD coagulation dissector or other additional endoscopic instruments. In analogy to EMR+ , the technique is termed “ESD+ ”.

ESD+ with the AWC has not been systematically evaluated in animal models or patients before. Here, we investigate the feasibility of this novel method in a pre-clinical porcine ex vivo animal model for the first time. To this end, we prospectively compare the novel technique ESD+ to conventional ESD in the clinically relevant sizes of 3 cm and 4 cm in antegrade as well as in retrograde positions in order to investigate, which lesions are particularly appropriate for the indication of ESD+.

## Materials and methods

The study was a prospectively designed ex vivo trial. Since no humans or living animals were included, it was exempt from IRB. We conducted the experiments at our Laboratory for Experimental Endoscopy in the Department of Gastroenterology and Gastrointestinal Oncology of the University Medical Center Goettingen in Germany.

We used cleaned and frozen porcine stomachs. They were defrosted and placed into the EASIE-R simulator (Erlangen Active Simulator for Interventional Endoscopy, Endosim, LLC, Hudson, MA, USA) prior to the procedure. This model has also been evaluated at our research unit for several endoscopic procedures [7, 8]. The EASIE-R simulator has become widely established in interventional training and endoscopic research [16, 17].

All interventions (ESD and ESD+) were performed by a well-trained endoscopist with previous experience in ESD technique in humans and in animal models.

### Preparation of the porcine stomach

Prior to intervention, we defined standardized flat lesions, measuring 3 cm or 4 cm in the porcine stomach by marking with coagulation dots. The lesions were applied either in antegrade or in retrograde position. After suture closure, the stomach was transferred into the EASIE-R model. Esophagus and stomach were then fixed to the plastic shell of the model [8, 17].

### ESD and ESD+ procedure

We conducted ESD and ESD+ with a gastroscope (EG-530D Fujinon, Fujifilm, Japan), the AqaNife 2.0 mm (Ovesco Endoscopy, Tuebingen, Germany) and, in cases of classical ESD, with an ESD cap (Olympus, Japan). We used Hydroxyethyl starch (HAES) as ESD injection fluid, mixed with methylene blue dye in order to optimize visualization and tissue differentiation. ERBE VIAO 200 (ERBE Elektromedizin, Tuebingen, Germany) was used as electrosurgical unit with EndoCut Q 1/1/1 setting.

**Fig. 1:**
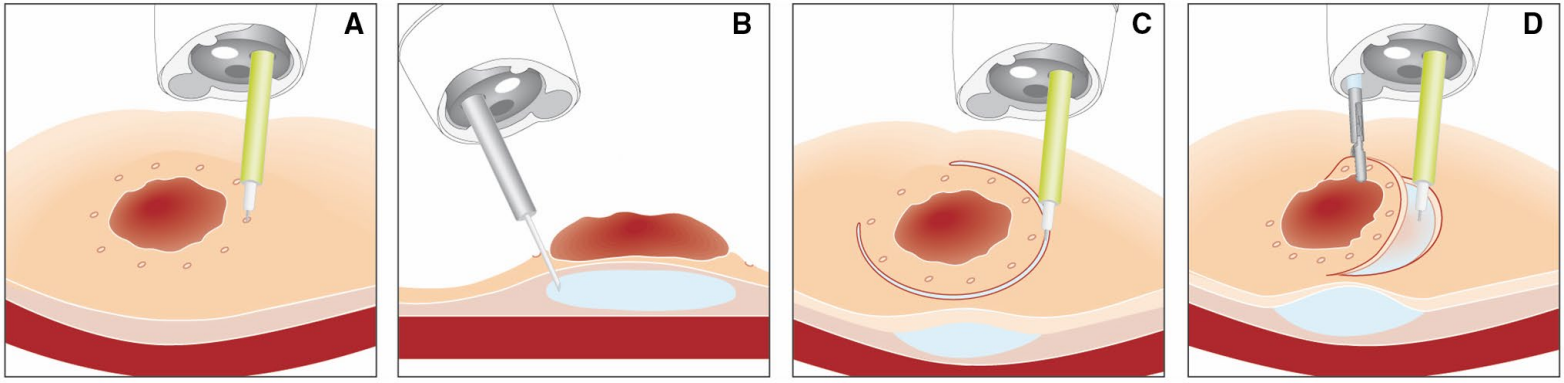
Principle of ESD+ procedure. **A** Target lesion, **B** submucosal injection of the target lesion, **C** circumferential ESD incision, **D** mobilization of the lesion’s flap with a grasper introduced via the AWC (Source: with permission from Ovesco Endoscopy AG, Tuebingen, Germany)

**Fig. 2:**
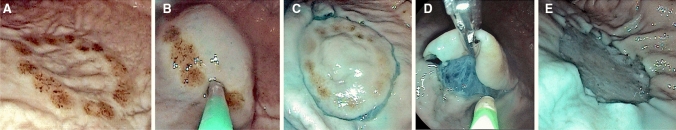
Application of ESD+ in the ex vivo model. **A** Target lesion, **B** submucosal injection of the target lesion, **C** circumferential ESD incision, **D** mobilization of the lesion´s flap with a grasper introduced via the AWC, **E** Post-resection site after ESD+

ESD+ procedures were performed with the AWC device (Ovesco Endoscopy, Tuebingen, Germany). Figure 1 shows the principle of ESD+ with the help of the AWC. In Fig. 2, the use of the ESD+ technique in the ex vivo model is illustrated.

### Additional working channel (AWC)

The principle of the AWC is shown in Fig. 1. It has a flexible attachment and a shaft with a length of 122 cm (endoscope insertion length: 103–110 cm). There is an adaptor for fixation at the endoscope handle with Luer-lock, a valve and a sleeve with adhesion tape. The AWC is suitable for the mounting on endoscopes with a diameter from 8.5 to 13.5 mm. The introduction of instruments with an outer diameter of up to 2.8 mm is possible [8]. We performed all AWC procedures with the AWC in the counterpart position to the working channel.

### Data collection

We recorded the following parameters by an independent observer: Prepared lesion´s size (3 cm or 4 cm), position of the lesion (antegrade or retrograde), time of ESD and ESD+ procedure (minutes), adverse effects (muscularis damage, perforations), rate of en bloc resection (“R0”).

Following every ESD and ESD+ , the resected specimens were spread out, pinned on cork plates and en bloc resection was evaluated and documented. Procedure time was defined from submucosal injection to complete resection of the lesion. Muscular injury was detected by visual evaluation of every resection site. Potential perforations were evaluated by an insufflation test of the porcine stomach.

### Statistical analysis

We performed data analysis using SPSS (IBM, Armonk, NY, USA). Chi-square-test was used for the analysis of muscularis damage/ perforation events. Mann–Whitney *U* test was used for the analysis of time of procedure. *P* values less than 0.05 were considered statistically significant and are marked by *.

## Results

**Fig. 3:**
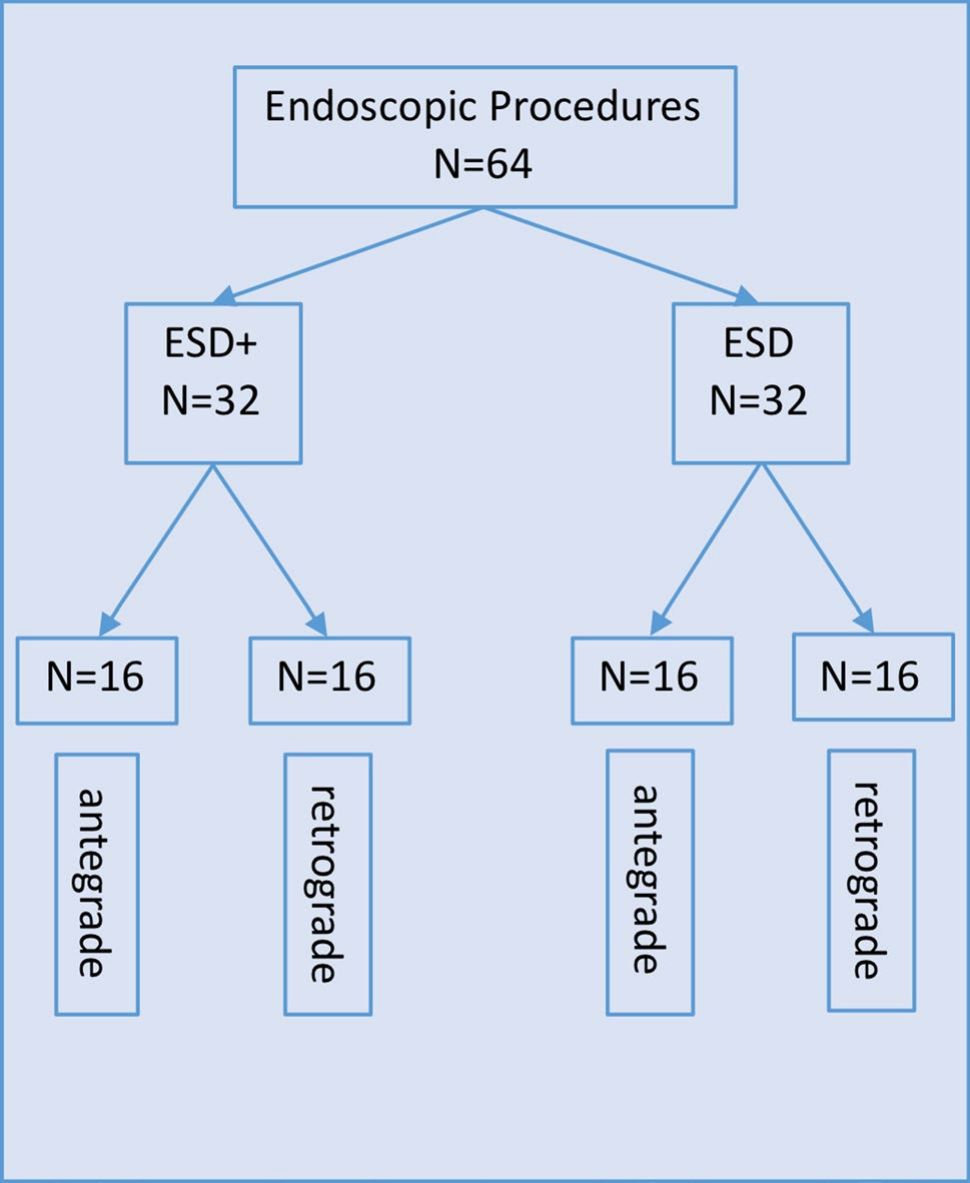
Study design

In total, 64 endoscopic procedures (32 ESD+ , 32 ESD) were performed by an experienced endoscopist in the ex vivo porcine model (Fig. 3).

In the ESD+ as well as in the ESD groups, lesions with two different sizes were set with a diameter of 3 cm (*n* = 16 per group) and 4 cm (*n* = 16 per group) (Fig. 3).

In every group, we prepared eight lesions in antegrade and eight lesions in retrograde position (overall 32 antegrade, 32 retrograde lesions) (Fig. 3).

Overall, nine stomachs were used, each with 6–9 lesions, dependent on stomachs´ and lesions´ sizes.

### Rate of en bloc resection

ESD+ as well as ESD fulfilled the demands of a secure resection in terms of an en bloc resection rate of both 100% in all lesions´ sizes (32/32; 100%).

### Time of procedure dependent on size (minutes)

**Fig. 4:**
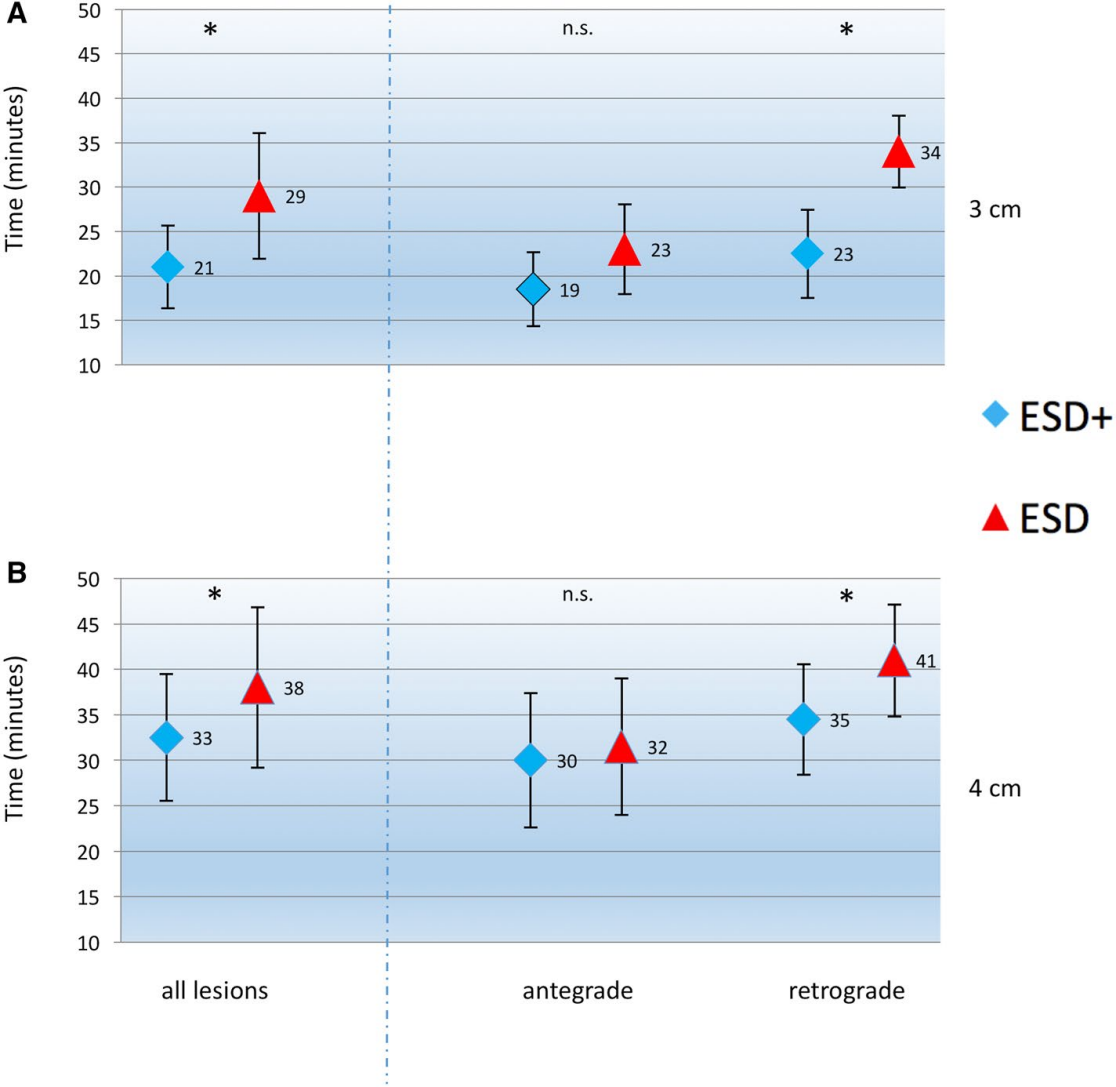
Time of procedure (minutes) dependent on the lesion´s position (antegrade/ retrograde). **A** 3 cm lesions, **B** 4 cm lesions

In both ESD+ and ESD, overall procedure time was significantly shorter in 3 cm lesions than in 4 cm (ESD+ 21.0 (SD: 4.7) vs. 32.5 (SD: 7.0) minutes, *p* = 0.01*; ESD 29.0 (SD: 7.0) vs. 38.0 (SD: 8.8) minutes, *p* = 0.03*) (Fig. 4A, B).

### Time of procedure dependent on technique (ESD+ vs. ESD, minutes)

Across all groups, ESD+ was significantly faster than ESD (24.5 min (SD: 7.8) vs. 32.5 min (SD: 9.0), *p* = 0.025*).

In 3 cm lesions, the time of procedure was significantly shorter with ESD+ compared to ESD (21.0 min (SD: 4.7) vs. 29.0 min (SD: 7.0), *p* = 0.006*) (Fig. 4A). Also in 4 cm lesions, ESD+ was significantly faster than ESD (32.5 min (SD: 7.0) vs. 38.0 min (SD: 8.8), *p* = 0.039*) (Fig. 4B).

### Time of procedure dependent on the lesion´s position (antegrade/ retrograde, minutes)

In 3 cm lesions, the median time of procedure with ESD+ in antegrade lesions was 18.5 min (SD: 4.1) compared to 23.0 min (SD: 5.1) with ESD (*p* = 0.223) (Fig. 4B). Thus, ESD+ was not significantly faster in antegrade 3 cm lesions.

In contrast, ESD+ was significantly faster in 3 cm retrograde lesions with a median of 22.5 min (SD: 5.0) compared to ESD (34.0 min (SD: 4.0) (*p* = 0,002*) (Fig. 4A).

In analogy to 3 cm, also in 4 cm lesions the median time of procedure with ESD+ in antegrade lesions did not significantly differ compared to ESD (30.0 min (SD: 7.4) compared to 31.5 min (SD: 7.5), *p* = 0.429) (Fig. 4B).

In retrograde lesions with 4 cm, ESD+ was again significantly faster with 34.5 min (SD: 6.1) compared to 41.0 min (SD: 6.2) with ESD (*p* = 0,011*) (Fig. 4B).

### Safety and adverse events

In the ESD groups, 0 perforations and 6 muscularis damages occurred (6/32, 18.75%) with 2 in 3 cm lesions (2/16, 12.5%) and 4 in 4 cm lesions (4/16, 25.0%). Of those, one occurred in an antegrade position and 5 occurred in retrograde positions.

Also in the ESD+ groups, there were 0 perforations. One muscularis damage occurred in an antegrade ESD+ lesion of 4 cm (1/32, 3.13%). In both techniques, the risk of muscularis damages increased with size, showing a total of 2 (2 in ESD and 0 in ESD+) muscularis damages in 3 cm lesions and 5 (4 in ESD and 1 in ESD+) in 4 cm lesions.

In both techniques, the risk of muscularis damages increased with the difficulty of the lesion´s position showing in total 2 (1 in ESD and 1 in ESD+) muscularis damages in antegrade positions and 5 (5 in ESD and 0 in ESD+) muscularis damages in retrograde lesions.

Thus, the rate of muscularis damages was significantly lower under ESD+ compared to ESD (1 vs. 6, *p* = 0.045*).

## Discussion

ESD+ is a combination of ESD with the grasp technique of the recently launched AWC. The aim of our study was to evaluate ESD+ in comparison with classical ESD for the first time.

Over the last decades, endoscopic resection techniques have evolved from EMR to ESD or endoscopic full-thickness resection (EFTR) with the full-thickness resection device (FTRD) in specific indications [18–20]. Many approaches have been tested to accelerate and secure endoscopic resections. For the purpose of achieving better intraluminal tissue traction, various endoscopic devices have been designed, including external forceps, magnetic anchors, clips with attached strings, rubber bands, the EndoLifter, and the creation of a pulley system with clips to facilitate endoscopic traction [15, 21–27]. However, the optimal traction device for endoluminal surgery has not been identified so far. For this reasons, further basic research is necessary in this field.

Today, in the hand of an endoscopic expert, ESD offers a reliable and anatomically convincing method for large resections with lower rates of recurrence and a higher rate of R0 resections compared to EMR [28]. Several meta-analyses compared EMR with ESD in terms of the treatment of early gastric cancer with higher en bloc resection rates, higher histologically complete resection rates and lower recurrence frequencies for ESD [13, 29–31]. However, the advanced technique of ESD has a long procedure time. It is challenging and technically complex with a long flat learning curve also for well-trained experienced endoscopists [13]. Furthermore, ESD is associated with a relevant rate of adverse events, especially perforations in up to 4–10% [32].

For these reasons, it is desirable to develop and clinically implement additional endoscopic tools in order to accelerate procedure times and to improve the feasibility and safety of ESD especially in challenging anatomic sites, e.g., lesions in retrograde endoscopic positions.

Against this background, ESD+ was recently launched. In analogy to EMR+ , it is based on the AWC [7, 9].

In EMR, a grasp and snare technique using a dual channel endoscope has already been described [33–35]. There is also data about the use of double-channel endoscopes in ESD [36, 37]. However, the practicability of a double-channel endoscope in ESD is even more limited than in EMR, especially in retrograde lesions. The close and fixed distance between the two working channels results in a lack of sufficient triangulation, flexibility, and overview. Moreover, a dual channel endoscope is an expensive investment for endoscopy units and is consequently not available in many endoscopy units. The AWC is mounted at the tip of a standard endoscope in analogy to the setup known from the FTRD [18], thus making a dual channel endoscope dispensable. In contrast to a dual channel endoscope, wider and more variable positions of both working channels (standard channel plus AWC) can be achieved by turning its cap [6]. This results in a better visibility and more flexible triangulation of the instruments if required.

The EndoLifter by Olypmus has also been a promising approach. However, the EndoLifter is limited to its grasping forceps fixed on a metal bracket and is only approved for gastric ESD. Compared to the EndoLifter, the AWC used in ESD+ is more flexible as it functions as a full-featured additional working channel with the opportunity of introducing various endoscopic instruments which can be independently applied to the intraluminal target.

First of all, our results represent the high reliability of ESD+ and ESD in both lesions´ sizes of 3 and 4 cm in terms of an en bloc resection rate of each 100%. As known from clinical everyday-life, procedure time rises with the lesion´s size and the difficulty of its position (e.g., antegrade vs. retrograde). This is recapitulated by our data in both sizes in ESD+ as well as in ESD.

Most importantly, in our setting, ESD+ renders preparation and resections faster. This becomes most evident in the more challenging retrograde lesions. This was significant as well in 3 cm as in 4 cm.

However, we found that the benefit of ESD+ was somewhat diminished in larger lesions and subsequent resection flaps, most likely due to fact the AWC cannot be simultaneously applied with an ESD cap.

Representing the clinical practice of ESD, our results show that the rate of muscular damages, and consequently potential perforations, principally rises with the size of the lesion. With regard to the technique´s safety, we observed significantly fewer muscularis damages of ESD+ compared to ESD. Consequently, ESD+ might enhance the safety of ESD especially in bigger or anatomical challenging lesions.

Our prospective trial was performed in an established and well-evaluated ex vivo animal model. However, there are some limitations concerning the transferability from the porcine ex vivo model to living humans. First, the porcine stomach shows a higher mucosal rigidity compared to the human gastric mucosa that affects the technical opportunities of ESD+ and ESD. Tissue movement, bleeding, histopathological evaluation and other physiological factors can obviously not be recapitulated in our ex vivo model. The resections were conducted by a single endoscopist leading to a good internal validity but coming along with a potential systematic bias. A potential disadvantage of ESD+ itself may be tissue damage that can principally occur to the specimen as a result of grabbing the lesion with the forceps via the AWC.

However, until now there is no ESD system commercially available that combines both the AWC and an ESD cap. We think that the development of a specific ESD cap is desirable to enable the simultaneous use of an ESD cap and the AWC in large lesions.

## Conclusion

The newly developed ESD+ technique with an additional working channel (AWC) facilitates faster and safer resections of flat gastric lesions compared to conventional ESD.

In an ex vivo porcine model, we could show that ESD+ works and increases the speed and safety of endoscopic resections. This particularly applies to the resection of lesions in challenging anatomic positions, e.g., in retrograde position.

As already shown in EMR+, the AWC device allows an easy transformation of a standard single-channel endoscope to double-channel functionality. This leads to a good opportunity of bimanual working by triangulation resulting in potentially better intraluminal resections accompanied by a more efficient tissue traction.

In our view, ESD+ could contribute to a safer applicability of ESD particularly in standard endoscopy units with endoscopists less familiar with ESD. However, this ought to be subject of further studies.

## Electronic supplementary material

Below is the link to the electronic supplementary material.

Supplementary file 1 (MP4 39,003 kb)

## Abbreviations

AWC: Additional working channel
EFTR: Endoscopic full-thickness resection
EMR: Endoscopic mucosal resection
EMR+: Endoscopic mucosal resection using the AWC
ESD: Endoscopic submucosal dissection
ESD+: Endoscopic submucosal dissection using the AWC
FTRD: Full-thickness resection device
HAES: Hydroxyethyl starch
IRB: Institutional Review Board (Ethikkommission)
R0: No residual tumor
SD: Standard deviation
